# Species Boundaries Between Three Sympatric Oak Species: *Quercus aliena, Q. dentata*, and *Q. variabilis* at the Northern Edge of Their Distribution in China

**DOI:** 10.3389/fpls.2018.00414

**Published:** 2018-03-29

**Authors:** Jia Lyu, Jia Song, Yuan Liu, Yuyao Wang, Junqing Li, Fang K. Du

**Affiliations:** Molecular Ecology Lab, College of Forestry, Beijing Forestry University, Beijing, China

**Keywords:** *Quercus*, cpDNA, nSSRs, gene flow, fine scale

## Abstract

Oaks are important timber trees with wide distributions in China, but few genetic studies have been conducted on a fine scale. In this study, we seek to investigate the genetic diversity and differentiation of three sympatric oak species (*Quercus aliena* Blume, *Quercus dentata* Thunb. ex Murray, and *Quercus variabilis* Blume) in their northern distribution in China using 17 bi-parentally inherited nSSRs markers and five maternally inherited chloroplast DNA (cpDNA) fragments. Both the cpDNA and the nSSRs show a high level of genetic differentiation between different oak sections. The chloroplast haplotypes are clustered into two lineages. Clear species boundaries are detected between *Q. variabilis* and either *Q. aliena* or *Q. dentata*. The sharing of chloroplast haplotype H1 between *Q. aliena* and *Q. dentata* suggests very recent speciation and incomplete lineage sorting or introgression of H1 from one species to another. The nSSRs data indicate a complete fixation of variation within sites for all three oak species, and that extensive gene flow occurs within species whereas only limited gene flow is detected between *Q. aliena* and *Q. dentata* and nearly no gene flow can be detected between *Q. aliena* and *Q. variabilis* and between *Q. dentata* and *Q. variabilis*. Prezygotic isolation may have contributed to the species boundaries of these three sympatric oak species.

## Introduction

Transfer of genetic material across closely related species is common, especially in coexisting taxa (Rieseberg and Wendel, 1993; Arnold, 1997; Rieseberg et al., 2006; Kim et al., 2008). Hence, resolving the amount and extent of species boundaries between closely related species is often challenging. In recent years DNA sequence analysis has been widely employed to examine the relationship between genetic variation and traditional morphological boundaries among closely related species (e.g., Jaramillo-Correa et al., 2008; Du et al., 2011 for gymnosperm-conifers; Arnold et al., 2012; Eaton et al., 2015 for angiosperms). These studies show that molecular polymorphisms for neutral markers are often widely shared between closely related species, and that fixed genetic differences may vary because of the genomes or genes studied (Petit and Excoffier, 2009).

Population genetics studies on oaks using nuclear DNA markers have shown that these species tend to have high levels of within-population genetic variation and generally low variation among populations because they are highly crossing and wind-pollinated long-lived forest trees (Hamrick and Godt, 1989; Kremer and Petit, 1993; Starck et al., 1993; Quang et al., 2008). Oaks are known to have a propensity for interspecific hybridization. Gene flow and introgression have been intensely studied in natural populations across Europe (e.g., Dumolin-Lapègue et al., 1999; Petit et al., 2002; Curtu et al., 2007; Salvini et al., 2009; Chybicki et al., 2012; Antonecchia et al., 2015; Fortini et al., 2015) and North America (e.g., Whittemore and Schaal, 1991; Dodd and Afzal-Rafii, 2004; Peñaloza-Ramírez et al., 2010; Moran et al., 2012). Genetic studies on oak species indicate that hybridization and introgression between sympatric oaks occur frequently, even between morphologically and ecologically distinct ones (Whittemore and Schaal, 1991; Dumolin-Lapègue et al., 1999; Petit et al., 2002). Not only large scale studies, but also studies on a fine scale have verified the occurrence of interspecific gene flow and introgression among different oak species (Salvini et al., 2009; Antonecchia et al., 2015).

Compared to Europe and North America, studies on *Quercus* in Asia are limited but an increasing number of studies have been reported in recent years, especially those taking a molecular perspective. A number of genetic studies have focused on genetic diversity and variation in closely related oak species; examples of such investigations include analyses of the genetic diversity of natural populations of *Q. mongolica* Fisch. ex Ledeb. (Li et al., 2003; Zhang et al., 2007), genetic diversity and differentiation along altitudinal gradients in *Q. crispula* Blume growing in Japan (Ohsawa et al., 2007), chloroplast polymorphism of *Q. serrata* Thunb. ex Murray, *Q. mongolica* Fisch. ex Ledeb. var. *crispula* (Blume) Ohashi, *Quercus dentata* Thunb. ex Murray, and *Quercus aliena* Blume in Japan, Korea, China, and Russia (Kanno et al., 2004; Okaura et al., 2007), geographic patterns of genetic variation in nuclear and chloroplast genomes of two related oaks (*Q. aliena* and *Q. serrata*) in Japan (San Jose-Maldia et al., 2017), differentiation of three closely related Japanese oak species and detection of interspecific hybrids using AFLP markers (Matsumoto et al., 2009) and exploring the species limits and dynamics of speciation in the two closely related Chinese oaks *Q. mongolica* and *Q. liaotungensis* Koidz. (Zeng et al., 2010, 2011). However, almost all the genetic studies carried out on *Quercus* in Asia have focused on genetic structure or phylogeography on a large scale; studies of gene flow on a fine scale have been relatively limited.

Various molecular markers have been used to investigate gene flow. Among them, nuclear microsatellite markers or Simple Sequence Repeats (nSSRs) which are bi-parentally inherited and transfer extensive gene flow through both pollen and seeds (Petit et al., 2005) are one of the most popular sources of molecular markers used in population genetic studies because of co-dominant inheritance, high degree of polymorphism and relative ease of transfer between closely related species (Guichoux et al., 2011a). The chloroplast markers show great capability for tracing long-term effects of hybridization and introgression in natural populations (Whittemore and Schaal, 1991) as they are predominantly maternally inherited and transfer only limited gene flow via seeds in most angiosperm plants (Mogensen, 1996).

In this study, we employed both bi-parentally inherited nSSRs markers and maternally inherited cpDNA fragments as molecular markers to estimate the genetic diversity and genetic differentiation of three sympatric oak species (*Q. aliena, Q. dentate*, and *Quercus variabilis* Blume), and gene flow among them in their northern distribution in China. All three oak species are widely distributed across China (Huang et al., 1999) and Beijing Municipality is at the northern edge of their sympatric distribution. *Quercus aliena* and *Q. dentata* belong to the white oak section *Quercus* (Hubert et al., 2014), and the two species differ mainly in leaf morphology (Supplementary Figure 1): leaves of *Q. aliena* have 1–1.3 cm petioles, while *Q. dentata* leaves have much shorter petioles (2–5 mm), with densely grayish brown stellate tomentose on the abaxial surface and usually are very big (c. 10–30 cm long, 6–30 cm wide). *Quercus variabilis* belongs to section *Cerris* (Hubert et al., 2014). Its leaves are ovate–lanceolate to narrowly elliptic, glabrous, with a 1–3 (−5) cm petiole and spiniform teeth on the margin (Huang et al., 1999, Supplementary Figure 1). Like other oaks, all the three oak species are diploid outcrossing species with wind-pollination (Kremer et al., 2007). The fine-scale study reported herein was conducted to address the following issues: (1) What are the levels of genetic diversity and genetic differentiation of the three oak species? (2) Is there any evidence for gene flow among these oak species? (3) If gene flow occurs, what is its scale and extent and what are the explanations for it?

## Materials and methods

### Study site and sample collection

Three sympatric oak species: *Q. aliena, Q. dentata*, and *Q. variabilis* were studied in their northern distribution, at Shangfang Mountain located in the southwest of Beijing Municipality, P. R. China (115°48′E, 39°39′N). Shangfang Mountain is a branch of the Taihang Mountains with fold-thrust geological structures. It has a mean altitude of 400 m and a summit of over 880 m. The mountain slopes range from 20 to 70°, forming very steep terrain with dozens of valleys. Within this region, hundreds of ancient Taoist and Buddhist temples scatter throughout the forest, with the oldest one being dated back to the Eastern Wei period (535 AD). The forest here is a well-preserved secondary forest in north China covering an area of approximately 340 hectares. Tree species of Shangfang Mountain mainly belong to *Quercus, Pinus*, and *Platycladus* with *Quercus* including mostly *Q. aliena, Q. dentata*, and *Q. variabilis* and a limited number of <20 individuals of *Q. mongolica*.

**Figure 1:**
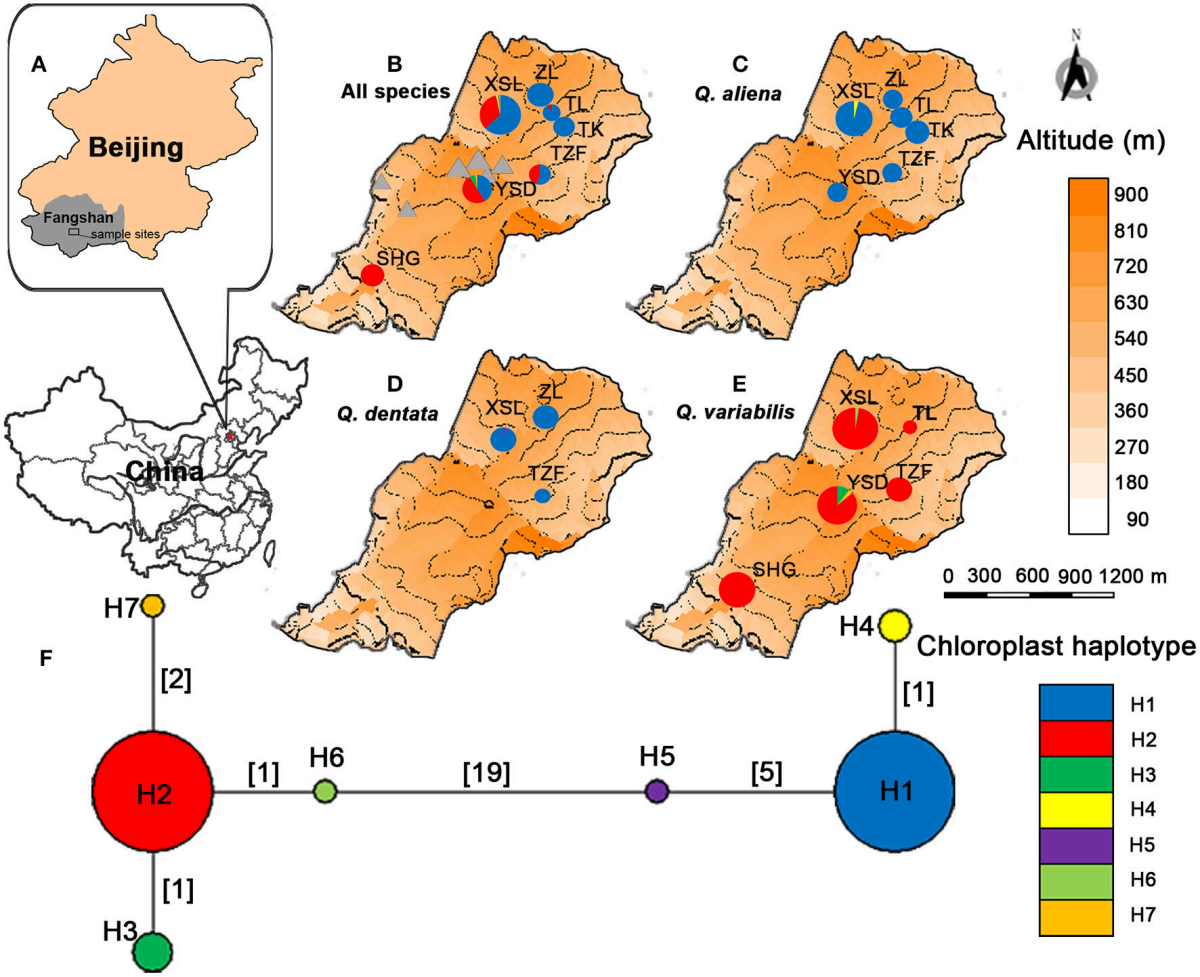
Localization of Shangfang Mountain (the gray rectangle) on the map of Fangshan district in Beijing Municipality, P.R.China **(A)**. Localization and cpDNA structure of the three sympatric oaks *Quercus aliena, Q. dentata* and *Q. variabilis*. Circle sizes reflect the numbers of individuals genotyped (*n* = 29–116). Triangles represent temples, village, caves and bridges in the sample sites. **(B)**. Localization and distribution of chloroplast haplotypes of *Q. aliena* **(C)**, *Q. dentata* **(D)**, *Q. variabilis* **(E)**. Inferred phylogenetic network of the seven cpDNA haplotypes found **(F)**. Each haplotype is represented by a circle whose size is proportional to its frequency across all species. Numbers in brackets on branches indicate the number of mutations separating haplotypes.

Leaf material was collected from seven 50 by 50 m sites in this region (Figure 1 and Supplementary Table 1). The sites were selected for each species on the basis of site accessibility and sample availability. In general, two leaves were collected from each mature tree of each of the three oak species provided that they were at least five meters apart and stored in silica gel for further analysis. In total, 432 individuals were collected including 207 *Q. aliena* (six sites), 68 *Q. dentata* (three sites), 156 *Q. variabilis* (five sites) and only one individual with intermediate morphology between *Q. aliena* and *Q. dentata* (at site TK). The latitude, longitude, and altitude of each individual sampled were recorded by GPS (Garmin, USA).

### DNA extraction

Genomic DNA was extracted from silica gel dried leaves using a plant genomic DNA kit (Tiangen Biotech, China) following the manufacturer's instructions. The quality of DNA was initially assessed by a 1.00% (w/v) agarose gel. Then, the quantity of DNA was measured by an ultramicro-spectrophotometer (Thermo Fisher, USA). Genomic DNA of each sample was adjusted to a final concentration of 20–30 ng/μL for subsequent use.

### Chloroplast DNA amplification and sequencing

For the chloroplast DNA (cpDNA) study, 14 individuals from the seven sites (at least one individual of each oak species present at each site) were initially used to identify polymorphisms with 13 pairs of universal primers described in Shaw et al. (2005, 2007) and Du et al. (2017) (see Supplementary Table 2 for details). Five pairs of cpDNA primers (*accD-psaI-75R, trnH-psbA, rpS12-rpL20, rpS16*, and *trnQ-trnS*) that exhibited polymorphisms in this first scan were subsequently used to analyze all 432 individuals, out of which 343 individuals with high quality were taken forward for further investigation. Each polymerase chain reaction (PCR) amplification mixture contained 20 ng of genomic DNA, 67 mM Tris-HCL, 16 mM ammonium sulfate, 100 μM of each of the four dNTPs, 2 mM MgCl_2_, 1 ng of BSA, 0.2% mercaptoethanol, 0.2 μM of each primer, and 0.2 units of *Taq* polymerase (Tiangen Biotech, China). The amplification was carried out using 1 cycle of 3 min at 94°C, 35 cycles of 30 s at 94°C, 45 s at 50 to 55°C, 45 s at 72°C, and 1 cycle of 10 min at 72°C as modified by Du et al. (2017). The PCR products were visualized on 2% agarose gels. Templates were sequenced in the reverse direction with five reverse primers. Sequencing was performed using BigDye Terminator V 3.1 (Applied Biosystems, USA) following the manufacturer's instructions. Samples were run on an ABI 3130 Genetic Analyzer (Applied Biosystems, USA). Sequences were checked using Chromas V 2.2 and only those of high quality giving single peaks were used in subsequent analysis. DNA sequence alignment was carried out using Clustal W in MEGA V6.0 (Tamura et al., 2013) with manual adjustments. Chloroplast sequences have been deposited in GenBank under the accession numbers of KY751544-KY751561.

### nSSRs amplification

Twenty-seven nSSRs primers reported by Dow et al. (1995), Steinkellner et al. (1997), Kampfer et al. (1998), Ueno and Tsumura (2008), Ueno et al. (2008, 2009), and Durand et al. (2010), were tested for their ability to identify polymorphic SSR (see Supplementary Table 3 for details). Each pair of primers was initially amplified for three individuals (one for each oak species), and the PCR products were visualized on 2% agarose gels. The successfully amplified templates were sequenced to confirm the existence of repeated motifs and to determine the numbers of repeats. Each primer revealing SSR motifs was then amplified for 12 individuals (four individuals for each oak species) to test for polymorphism in simplex PCR reactions using the M13-tail technique (Schuelke, 2000). In brief, three primers were synthesized for each genotyping experiment: a 5′ M13-tailed forward primer, a reverse primer and a fluorescently labeled M13 primer carrying a FAM, HEX, TAMRA or ROX label (Sangon Biotech, China). The PCR reaction mixture contained 1 × Taq buffer, 0.2 mM dNTPs, 10–20 ng template DNA, 1.6 pmol of the reverse primer, 1.6 pmol of a single fluorescently labeled M13 primer, 0.4 pmol of the forward primer and 1 U Taq polymerase (Tiangen Biotech, China). The following conditions were used for PCR amplification: 8 min initial denaturation at 95°C and followed by 30 cycles of 30 s denaturation at 95°C, a 30 s annealing step at 56°C, a 30 s elongation step at 72°C, and a final extension step at 72°C for 8 min as modified by Xu et al. (2013). nSSRs with a satisfactory number of alleles (>5) and good amplification quality were selected. In total, 19 pairs of nSSRs primers (GOT021, PIE227, FIR026, QmC00716, POR017, QmC00196, QmC00963, GOT011, MSQ13, FIR015, PIE163, WAG066, DN950726, Qmc00932, DN950446, Qmc02052, PIE271, WAG068, and CcC00063; see details in Supplementary Table 3) were selected for genetic diversity analysis of all individuals. 0.5 μL samples of the PCR products obtained, combined with each of the four different fluorescently labeled primers, were added to 10 μL formamide and 0.5 μL of LIZ standard (Applied Biosystems, USA) and the mixture was analyzed using an ABI 3730 Prism Genetic Analyzer (Applied Biosystems, USA). Alleles were scored using the software GeneMarker V2.2 (Softgenetics, USA). Each genotype was checked by two readers, as described by Guichoux et al. (2011b). When an allele was found by only one reader, the inconsistency was classified as type A; when the two readers detected different alleles for the same sample, the inconsistency was classified as type B. The readers tried to determine a consensus genotype. If consensus result could not be achieved, the data were treated as missing.

### Data analysis

#### cpDNA sequence analysis

A set of combined sequences (haplotypes) was constructed for all the individuals we analyzed. The haplotype diversity parameters were calculated by Permut 2.0 (Pons and Petit, 1996; Burban et al., 1999); these included the total genetic diversity (*H*_T_), the genetic diversity within populations (*H*_S_), the interspecies differentiation (*G*_ST_) and the interspecies differentiation taking similarities between haplotypes into account (*N*_ST_). Comparisons between *G*_ST_ and *N*_ST_ were carried out with 1,000 random permutations of haplotype identity. Haplotype frequency was calculated and graphed for each species and sampling site. Analysis of molecular variance (AMOVA) was performed using Arlequin V3.5 (Excoffier and Lischer, 2010). The number of permutations for significance test was set at 1,000 for all analyzes. Relationships between haplotypes were identified using NETWORK 5.0 with the median-joining model (Bandelt et al., 1999).

#### Microsatellite analysis

Null alleles were initially detected by Micro-Checker V2.2 (Oosterhout et al., 2004) and primers revealing null alleles were excluded from further analysis. The Bayesian model-based clustering program STRUCTURE V2.3 (Pritchard et al., 2000) was used to assign individuals to *K* species on the basis of genotypes. The number of clusters (*K*) was set to 1–10. The program was set to run 1,000,000 Markov Chain Monte Carlo iterations (MCMC), following 100,000 burn-in iterations without any species identification information (USEPOPINFO = 0). The best *K*-value was evaluated using mean LnP(K) and Δ*K* (Evanno et al., 2005) by the web-based program STRUCTURE HARVESTER (Earl and vonHoldt, 2012). To determine whether there were any hybrids, the admixture coefficient (*Q*) values were analyzed. The *Q*-value of purebreds should near to 0 or 1 and that of F_1_ hybrids should be close to 0.5. Based on our results and literature references, threshold values of *Q* ≤ 0.9 (Lepais et al., 2009; Peñaloza-Ramírez et al., 2010) and *Q* ≤ 0.8 (Labeyrie et al., 2014) were set to distinguish hybrids from purebreds. Individuals with *Q* ≥ 0.8 or 0.9 were considered to be purebreds, with *Q* < 0.8 or 0.9 from two genetic groups were considered to be hybrids between two oak species. We also conducted a Principal Coordinates Analysis (PCoA) to cross-validate the results of STRUCTURE using GenAlEx version 6.5 (Peakall and Smouse, 2012). The expected heterozygosity (*H*_e_), observed heterozygosity (*H*_o_), and the observed number of alleles (*N*_a_) were estimated by GenAlEx V6.5 (Peakall and Smouse, 2012). The Arlequin software package V3.5 (Excoffier and Lischer, 2010) was used to analyze molecular variance (AMOVA). As wind-pollinated species, oaks rely predominantly on pollen for gene exchange. The extent and direction of historical gene flow between species were estimated from nSSRs data using the program Migrate-n V3.6 (Beerli and Felsenstein, 2001; Beerli, 2006) by calculating the parameters θ (four times effective population size multiplied by mutation rate per site per generation) and *M* (immigration rate divided by the mutation rate). A continuous Brownian motion model and the default *F*_ST_ was used to generate initial theta and migration values. Next, five independent MCMC chains, each with 5, 000, 000 generations were set to run. We sampled every 100 steps under a constant mutation model, discarding the first 10,000 records as burn-in. The mode and 95% highest posterior density were then estimated after checking for convergence. Ten different models (Supplementary Table 4) with different direction and amount of gene flow among the three oak species were defined and the best model was selected by comparing the marginal likelihoods using thermodynamic integration (Bezier) in Migrate-n (Beerli and Palczewski, 2010).

## Results

### cpDNA diversity and differentiation

A total of 3,344 bp from five cpDNA fragments were sequenced and a matrix of combined sequences was constructed. Twenty substitutions and six indels were detected in 343 individuals and were combined into seven chloroplast haplotypes (H1-H7) (Supplementary Table 5). H1 was the most common haplotype and was found in most *Q. aliena* (175 out of 178) and *Q. dentata* (47 out of 48), and in the single individual with intermediate morphology. H2 was the dominant haplotype in *Q. variabilis*, being found across all sites. H3 and H7 were restricted to *Q. variabilis* at site YSD. H4, H5, and H6 were only found at site XSL in *Q. aliena, Q. dentate*, and *Q. variabilis*, respectively (Table 1, Figure 1).

**Table 1:** The proportions of cpDNA haplotypes and genetic diversity estimates for nSSRs within each oak species.

**Species**	**Sites**	**cpDNA**							**nSSRs**				
		**N**	**H1**	**H2**	**H3**	**H4**	**H5**	**H6**	**H7**	**N**	***N*_a_**	***H*_o_**	***H*_e_**
*Q. variabilis*	SHG	30	–	30	–	–	–	–	–	33	6.06	0.59	0.59
	TL	2	–	2	–	–	–	–	–	18	5.06	0.56	0.56
	TZF	16	–	16	–	–	–	–	–	19	5.35	0.53	0.55
	XSL	40	–	39	–	–	–	1	–	48	7.59	0.62	0.64
	YSD	29	–	25	3	–	–	–	1	31	5.94	0.58	0.57
*Q. aliena*	TK	39	39	–	–	–	–	–	–	37	9.12	0.67	0.65
	TL	27	27	–	–	–	–	–	–	40	8.77	0.68	0.66
	TZF	19	19	–	–	–	–	–	–	20	7.18	0.68	0.66
	XSL	53	51	–	–	2	–	–	–	54	9.47	0.69	0.66
	YSD	20	20	–	–	–	–	–	–	27	7.41	0.64	0.64
	ZL	20	20	–	–	–	–	–	–	22	7.82	0.69	0.68
*Q. dentate*	TZF	1	1	–	–	–	–	–	–	1	1.77	0.77	0.38
	XSL	23	22	–	–	–	1	–	–	23	7.65	0.68	0.64
	ZL	24	24	–	–	–	–	–	–	41	9.53	0.71	0.71
Total		343	223	112	3	2	1	1	1	414	–	–	–

*N, number of individuals; H, chloroplast haplotype; N_a_, No. of alleles; H_o_ and H_e_, observed and expected heterozygosity*.

These haplotypes were clustered into two lineages separated by at least 19 mutations (Figure 1). H1, H4, and H5 formed a lineage containing the white oaks (*Q. aliena* and *Q. dentata*) and H2, H3, H6, and H7 formed the second lineage, that of *Q. variabilis* (Figure 1). Within the white oak lineage, the relationship between H1 and H5 was more distant with five mutation steps separating them; H4 differed from H1 by one mutation step. In contrast, in the *Q. variabilis* lineage, the four haplotypes were closely related: H3 and H6 were separated by one mutation from H2; H7 differed from H2 with two mutation steps.

The total haplotype diversity was high (*H*_T_ = 0.51) and more than ten times greater than the genetic diversity within populations (*H*_S_ = 0.03; Table 2). The *N*_ST_ value was significantly higher than the *G*_ST_ value (*p* < 0.05), suggesting the existence of phylogeography structure (Table 2). The cpDNA diversity of *Q. variabilis* was the highest among the three oak species, followed by that of *Q. dentata* and *Q. aliena* (Table 2). The results of AMOVA among the three species revealed that 99.83% of the variation could be attributed to among-species diversity and 0.17% to within-species diversity (Table 3). The variation between *Q. aliena* and *Q. variabilis* was similar to that between *Q. dentata* and *Q. variabilis* (Table 3) which was nearly fixed between species. In contrast, the variation between *Q. aliena* and *Q. dentata* was largely harbored within species (Table 3). In the intraspecies analysis, AMOVA results indicated a high level of genetic variation within sites while negligible variation among sites (negative estimates for *Q. aliena* and *Q. dentata*; 0.01 for *Q. variabilis*, Table 3).

**Table 2:** Genetic diversity estimates for cpDNA and nSSRs markers in the oaks investigated.

**Species**	**cpDNA**					**nSSRs**			
	**N**	***H*_T_**	***H*_S_**	***G*_ST_**	***N*_ST_**	**N**	***N*_a_**	***H*_o_**	***H*_e_**
*Q. aliena*	178	0.01	0.01	0.02	0.02	200	12.29	0.68	0.67
*Q. dentata*	48	0.03	0.03	−0.00	0.00	65	10.77	0.70	0.71
*Q. variabilis*	117	0.07	0.06	0.06	0.04	149	9.18	0.59	0.63
All species	343	0.51	0.03	0.94	1^*^	414	7.05	0.65	0.61

N, number of individuals; H_T_, total genetic diversity; H_S_, genetic diversity within populations; G_ST_, interspecies differentiation; N_ST_, interspecies differentiation taking similarities between haplotypes into account;

* *, means N_ST_ is significantly larger than G_ST_ (p < 0.05); N_a_, No. of alleles; H_o_ and H_e_, observed and expected heterozygosity*.

**Table 3:** AMOVA results for cpDNA and nSSRs markers.

**Source of variation**	**cpDNA**					**nSSRs**				
	***df*^†^**	**SS^ζ^**	**VC^ω^**	Variation (%)^*^	**Mean *F*_ST_**	***df*^†^**	**SS^ζ^**	**VC^ω^**	Variation (%)^*^	**Mean *F*_ST_**
Among three species	2	6, 309.59	30.93	99.83	0.998	2	755.48	1.47	20.63	0.21
within species	340	17.69	0.05	0.17		825	4, 657.7	5.65	79.37	
Between *Q. aliena* and *Q. dentata*	1	0.14	0	2.71	0.03	1	148.48	0.73	11.13	0.11
within species	224	9.8	0.04	97.29		528	3, 065.72	5.81	88.87	
Between *Q. aliena* and *Q. variabilis*	1	5, 782.93	40.96	99.92	0.999	1	581.42	1.69	23.24	0.23
within species	293	9.86	0.03	0.08		696	3, 876.92	5.57	76.76	
Between *Q.dentata* and *Q. variabilis*	1	2, 776.93	40.79	99.76	0.998	1	329.45	1.79	24.31	0.24
within species	163	15.71	0.1	0.24		426	2, 372.77	5.57	75.69	
***Q. aliena***
Among sites	5	0.05	0	−0.18	0	5	48.29	0.06	1.07	0.01
within sites	172	1.93	0.01	100.18		394	2, 236.64	5.68	98.93	
***Q. dentata***
Among sites	2	0.18	−0.01	−3.89	−0.04	2	25.38	0.22	3.53	0.04
within sites	45	7.65	0.17	103.89		127	755.41	5.95	96.47	
***Q. variabilis***
Among sites	4	0.35	0	1.44	0.01	4	91.67	0.31	5.69	0.06
within sites	112	7.53	0.07	98.56		293	1500.31	5.12	94.31	

† df, degree of freedom;

ζ SS, sum of squares;

ω VC, variance components;

* *P < 0.05*.

### nSSRs diversity, genetic structure, and gene flow

Two nSSRs loci (QmC00196 and WAG066) presenting null alleles detected by Micro-Checker and 18 individuals showing poor amplifications were excluded from further analysis. In total, genetic diversity of 414 individuals was estimated based on 17 loci. All 17 nSSRs loci were polymorphic with the mean number of different alleles per locus (*N*_a_) varying from 9.18 to 12.29 (Table 2). In contrast to the results of cpDNA analysis, nSSRs data indicated that the genetic diversity of *Q. variabilis* was the lowest among the three oak species (*H*_e_ = 0.63; Table 2). The observed heterozygosity (*H*_o_) ranged from 0.59 (*Q. variabilis*) to 0.70 (*Q. dentata*) (Table 2). Within species, the *N*_a_ of *Q. variabilis* ranged from 5.06 (site TL) to 7.59 (site XSL). The expected heterozygosity (*H*_e_) ranged from 0.55 (site TZF) to 0.64 (site XSL), and the *H*_o_ from 0.53 (site TZF) to 0.62 (site XSL) (Table 1). The *N*_a_ of *Q. aliena* ranged from 7.18 (site TZF) to 9.47 (site XSL). The highest *H*_e_ was 0.68 at site ZL and the highest *H*_o_ was 0.69 at sites ZL and XSL (Table 1). Both *N*_a_ (9.53) and *H*_e_ (0.71) for *Q. dentata* had their highest values at site ZL and the *H*_o_ ranged from 0.68 (site XSL) to 0.77 (site TZF) (Table 1). The results of the overall AMOVA suggested that most of the variation occurred within species (79.4%; *F*_ST_ = 0.21), and only 20.6% of the variation was due to interspecies differences (Table 3). AMOVA results of the three species pairs (*Q. aliena* and *Q. dentata*; *Q. aliena* and *Q. variabilis*; *Q. dentata* and *Q. variabilis*) revealed a high level of genetic variation within species (Table 3). The intraspecies analysis indicated a high level of genetic variation within sites (Table 3).

**Figure 2:**
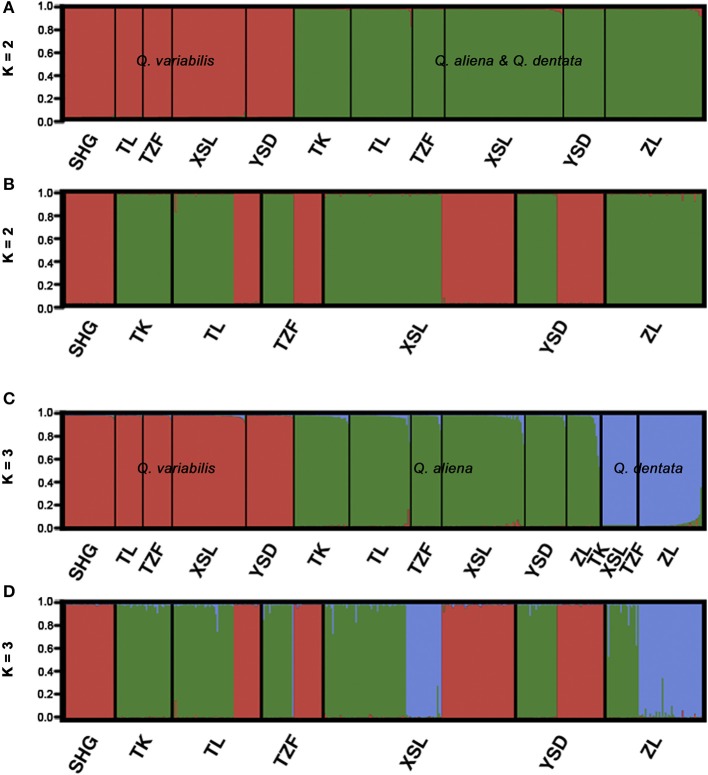
Genetic assignment for the three sympatric oaks based on variation at 17 microsatellite loci. Clustering of individuals into two **(A)** and three **(C)** different genetic clusters with decreasing admixture coefficient *Q*. Clustering of individuals into two **(B)** and three **(D)** different genetic clusters. Black lines separating different sites.

**Figure 3:**
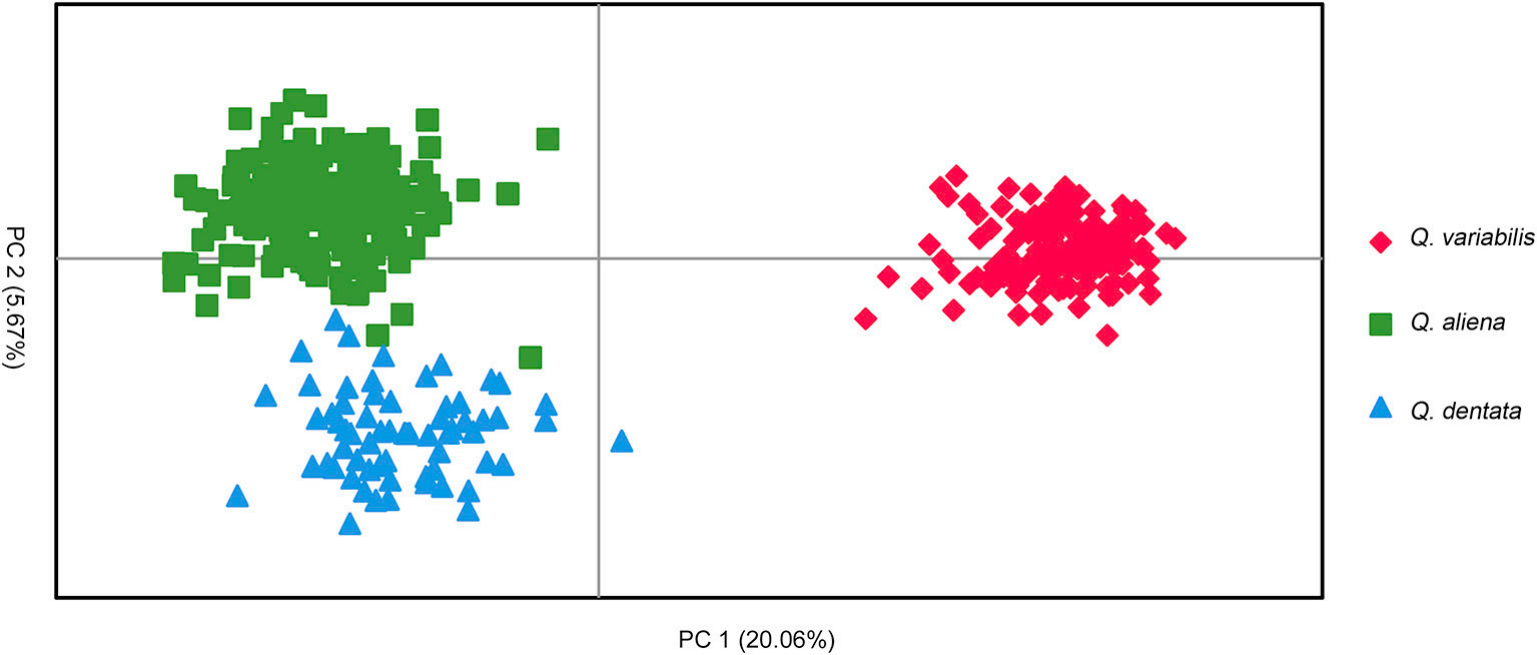
PCoA result for the three oak species.

The likelihood of classifying the data increased greatly from *K* = 1 to *K* = 3 and then increased slightly from *K* = 3 to *K* = 4, where it reached a plateau (Supplementary Figure 2). The Δ*K* (Δ*K* = 4157.04 when *K* = 2, Supplementary Figure 2) indicated that two was the optimal number of clusters (*K* = 2). However, the statistics also gave some support for *K* = 3 (Supplementary Figure 2). When *K* equaled to 2, one cluster corresponded to *Q. variabilis* (red), and the other one included *Q. aliena* and *Q. dentata* (green). When *K* equaled to 3 (Δ*K* = 1280.58), each species was classified into a distinct cluster with red, green and blue representing *Q. variabilis, Q. aliena* and *Q. dentata*, respectively (Figure 2). The PCoA result showed three distinct clusters, corresponding to *Q. aliena, Q. dentata*, and *Q. variabilis*. The first axis separated *Q. variabilis* from a cluster of *Q. aliena* and *Q. dentata*, and the second axis separated *Q. dentata* from *Q. aliena* and *Q. variabilis* (Figure 3).

**Figure 4:**
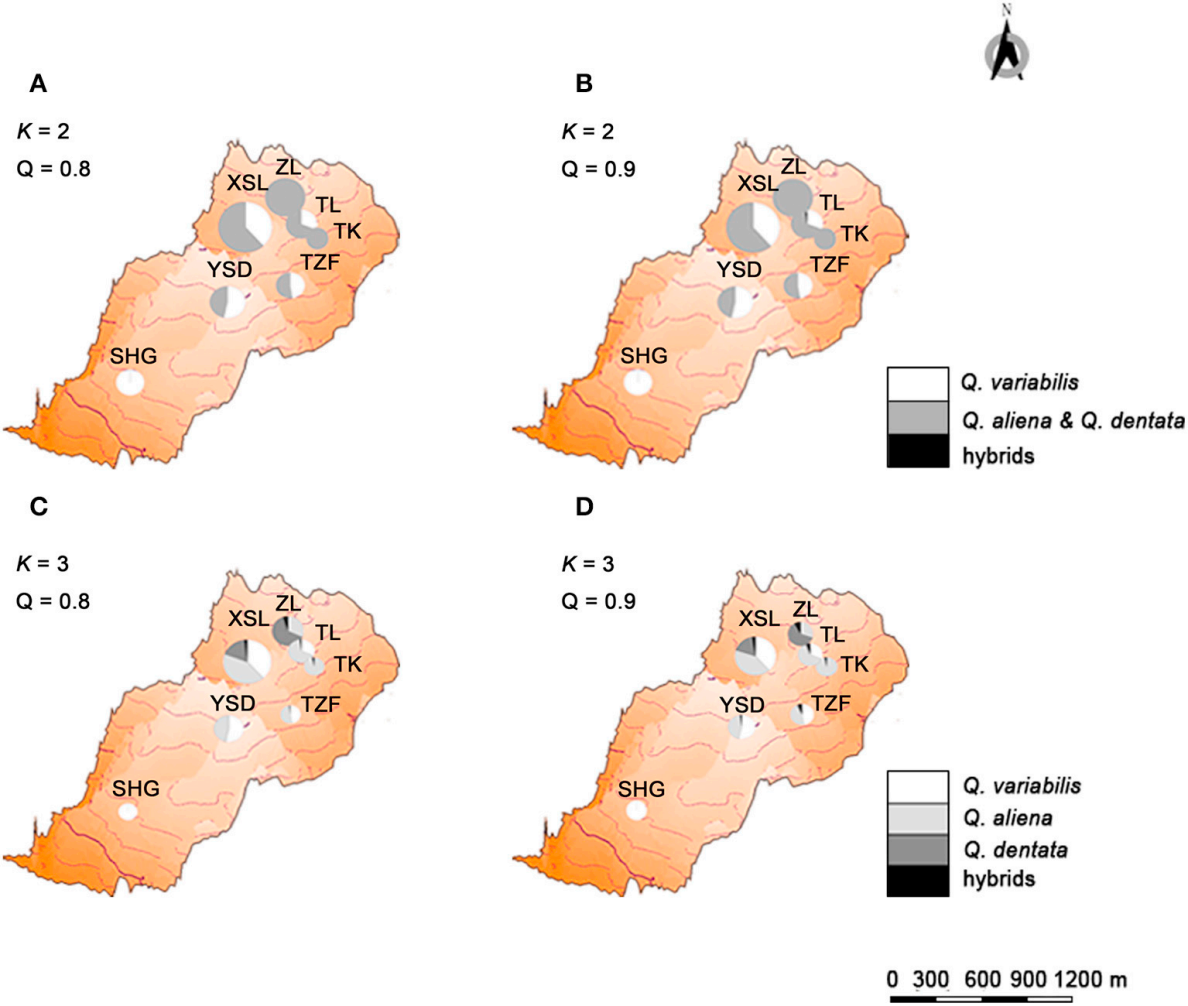
Geographical location and genetic structure of the three sympatric oaks. Geographical location and genetic structure of the two genetically assigned clusters of oaks when trees with *Q* < 0.8 **(A)** and *Q* < 0.9 **(B)** from two genetic groups were considered to be hybrids between two oak species. Geographical location and genetic structure of the three genetically assigned clusters of oaks when trees with *Q* < 0.8 **(C)** and *Q* < 0.9 **(D)** from two genetic groups were considered to be hybrids between two oak species.

If the threshold value of *Q* was set to 0.8, when *K* = 2, no individual was identified as a hybrid (Figure 2); when *K* = 3, seven individuals were hybrids between *Q. aliena* and *Q. dentata* with *Q*-values ranging from 0.24 to 0.46. These included the individual with intermediate morphology at site TK, one morphologically inferred *Q. aliena* at TL, two (one *Q. aliena* and one *Q. dentata*) at XSL and three (two *Q. aliena* and one *Q. dentata*) at site ZL. Hence, hybrids accounted for 1.7% of the total samples with only one individual being inferred to be an F1 hybrid (*Q*-value was 0.464, at site ZL) (Figures 2, 4).

If the *Q-*value was set to 0.9, when *K* = 2, only one individual at site TL was identified as a hybrid between *Q. aliena* and *Q. variabilis*, with a *Q*-value of 0.163. When *K* = 3, 14 individuals, including the individual with intermediate morphology at site TK, were considered to be hybrids. Among them, one hybrid between *Q. aliena* and *Q. variabilis* and six hybrids between *Q. aliena* and *Q. dentata* were identified with *Q*-values from 0.1 to 0.2 and another seven hybrids between these two species were detected with *Q*-values from 0.2 to 0.5; only one of these seven was considered to be an F1 hybrid (*Q*-value of 0.46, at site ZL) (Figures 2, 4).

A comparison of different models calculated by Migrate-n based on Bayesian factor values showed that model 2, with three population sizes and two migration rates (from *Q. aliena* to *Q. dentata* and from *Q. dentata* to *Q. aliena*) was the best model for evaluating the historical gene flow (Chen et al., 2017) among the three oak species. This model verified the occurrence of bidirectional historical gene flow between *Q. aliena* and *Q. dentata* (Table 4, *Q. aliena*→*Q. dentata, M* = 56.3; *Q. dentata*→*Q. aliena, M* = 57.0).

**Table 4:** Gene flow among the three species of oak estimated by Migrate-n.

		***M* (*m*/μ)**		
	θ	***Q. variabilis**→*	***Q. aliena**→*	***Q. dentata**→*
*Q. variabilis*	**0.1** [0.096–0.100]			
*Q. aliena*	**0.1** [0.096–0.100]			**57.0** [36.000–77.333]
*Q. dentata*	**0.1** [0.096–0.100]		**56.3** [34.667–77.333]	

*The mode of the posterior distribution is shown in bold and the values in square brackets give the 95% credibility interval; θ, 4Neμ; →, source populations; M, mutation-scaled immigration rate; m, immigration rate; μ, mutation rate*.

## Discussion

### Comparing genetic diversity and differentiation revealed by cpDNA data and nSSRs data

The analysis using cpDNA reveals low intraspecies diversity and high interspecies diversity in the oak complex on Shangfang Mountain, whereas both inter- and intraspecific diversity revealed by nSSRs data are high (Tables 1, 2). Sharing of haplotype H1 between *Q. aliena* and *Q. dentata* (Table 1) suggests very recent speciation and incomplete lineage sorting or introgression of H1 from one species to another. Sharing of cpDNA haplotypes has also been reported in other sympatric distributed *Quercus* species (Whittemore and Schaal, 1991; Dumolin-Lapègue et al., 1999; Kanno et al., 2004; Okaura et al., 2007). The inter- and intraspecific diversity revealed by nSSRs data (Tables 1, 2) are consistent with other oak species studied on a fine scale (Antonecchia et al., 2015).

For cpDNA, AMOVA results show high genetic differentiation among the three oak species and between the two sections of oak species. Variation between the combination of *Q. variabilis* and either *Q. aliena* or *Q. dentata* is completely fixed between species whereas most of the variation between *Q. aliena* and *Q. dentata* is within species (Table 3). For nSSRs, The AMOVA results show the majority of variation occurs within species (Table 3). The inconsistencies between the AMOVA results for cpDNA and nSSRs markers for all species, *Q. aliena* & *Q. variabilis* and *Q. dentata* & *Q. variabilis* agree with those reported by Petit et al. (2005), which can be explained by the different modes of inheritance of cpDNA and nuclear DNA. cpDNA is maternally inherited dispersing limited gene flow through seeds while nuclear DNA is bi-parentally inherited and can mediate extensive gene flow via both seeds and pollen. However, the AMOVA results for *Q. aliena* and *Q. dentata* indicate that interspecific differences are lower in cpDNA (Mean *F*_ST_ = 0.03 for cpDNA; *F*_ST_ = 0.11 for nSSRs) which may due to large proportion of individuals sharing haplotype H1 between the two species. Our results indicate chloroplast haplotypes are less species-specific than nuclear markers, probably because chloroplast DNA is more susceptible to drift than is nuclear DNA (Whittemore and Schaal, 1991; Dumolin-Lapègue et al., 1999; Petit and Excoffier, 2009).

For each species the intraspecific variation within sites is very high, with almost no genetic differentiation among sites within species for either cpDNA or nSSR markers (Table 3). These results indicate that even the rugged mountain ranges in this region have not impeded extensive gene flow mediated by seeds and pollen among sites which has helped to maintain the species integrity. Human modifications of the landscape such as the building of ancient temples, caves and bridges may have fragmented the forest, decreasing habitat size and increasing isolation between habitats and populations (Young et al., 1996; Su et al., 2003). However, for outcrossing and wind-pollinated tree species such as oaks, the fragmented spatial organization may not impede the genetic exchange via pollen among sites (e.g., reviewed by Herrera-Arroyo et al., 2013), as demonstrated here.

### Interspecific gene flow

The genetic assignment by STRUCTURE using nSSRs shows that two clusters are optimal, reconfirming the taxonomic classifications of these three oak species. The two white oaks were classified into one cluster as they are more closely related to each other than to *Q. variabilis*. Given that *K* = 3 is more biologically representative of the oak species sampled, we also discuss the admixture results for three clusters. With three genetic clusters, *Q. aliena, Q. dentata*, and *Q. variabilis* are all clearly separated (Figure 2). Low frequency of bi-directional gene flow between *Q. aliena* and *Q. dentata* were visually detected in STRUCTURE bar plots (Figure 2). These results are also confirmed by the PCoA results (Figure 3). The existence of reproductive barrier(s) between these two closely related species may be one of the explanations for the limited gene flow between them. At our study site, the flowering periods of *Q. aliena* and *Q. dentata* are asynchronous but they occur in fairly rapid succession. Moreover, the relative abundance of parental species and pollen discrimination can contribute to pre-pollination barriers, limiting interspecific pollen transfer, since abundant species are proportionally less involved in hybridization than minority species (Lepais et al., 2009, 2013; Salvini et al., 2009). The relative abundance of *Q. aliena* is more than twice of that of *Q. dentata* in our study sites, so interspecific gene flow may be more frequent in the core ranges of *Q. dentata* (Figure 2 shows more hybrids at site ZL than at site XSL). However, because neither seeds nor seedlings have been sampled in this study, we cannot rule out the existence of postzygotic isolation mechanisms such as seed abortion, hybrid lethality, weakness and sterility.

It should be noted, however, the high number of immigrants per generation 4Nm (calculated by θM, higher than one) detected by Migrate-n in *Q. aliena* and *Q. dentata*, which suggests the existence of relatively high level of symmetric historical gene flow between them (Table 4). These results are consistent with genetic differentiation based on cpDNA with the mean *F*_ST_ being 0.03 and 0.11 for cpDNA and nSSRs, respectively (Table 3). This will have to be borne in mind when attempting to interpret the species boundaries.

For either *K* = 2 or *K* = 3, only one individual was identified as a hybrid between *Q. aliena* and *Q. variabilis* (*Q* = 0.16) and no hybrids between *Q. dentata* and *Q. variabilis* were detected (Figure 2), a finding which suggests that there is almost no gene flow either between *Q. aliena* and *Q. variabilis* or between *Q. dentata* and *Q. variabilis*. Nearly no gene flow was detected between *Q. variabilis* and either *Q. aliena* or *Q. dentata* by Migrate-n (Table 4). Combining the cpDNA and nSSRs results, clear species boundaries between the two sections of oak on Shangfang Mountain were detected.

## Conclusion

In our study, we conducted a fine-scale exploration of genetic diversity, differentiation and gene flow in *Q. aliena, Q. dentata*, and *Q. variabilis*. The cpDNA results show high genetic differentiation between different oak sections. The nSSRs data indicate complete fixation of variation within sites for all three oak species and extensive gene flow occurs within species whereas interspecific gene flow between *Q. aliena* and *Q. dentata* is limited and almost no gene flow was detected between either *Q. aliena* and *Q. variabilis* or *Q. dentata* and *Q. variabilis*. The reasons for the low level of genetic exchange between *Q. aliena* and *Q. dentata* may include reproductive isolation, such as differences in the flowering time and in the relative abundance of parental species. Samples of seeds and seedlings should be included in future studies in order to gain a comprehensive understanding of the mechanism of genetic exchange; for example, to determine whether postzygotic reproductive isolation is the reason for the species boundaries of the three oak species in this area (Widmer et al., 2009).

## Author contributions

FD and JuL designed the research. FD, JiL, JS, YL, and YW collected the samples. JiL, JS, and YL performed the experiments and analysis. FD and JiL wrote the manuscript. All authors revised the manuscript.

### Conflict of interest statement

The authors declare that the research was conducted in the absence of any commercial or financial relationships that could be construed as a potential conflict of interest.
