# Supplementary material for: Species Boundaries Between Three Sympatric Oak Species: Quercus aliena, Q. dentata, and Q. variabilis at the Northern Edge of Their Distribution in China
Source: Front Plant Sci. 2018 Mar 29;9:414. doi: 10.3389/fpls.2018.00414 (PMC5890165; doi:10.3389/fpls.2018.00414)
Supplement: Supplementary file 1 [file Table1.docx]

Supplementary Material

**Species boundaries between three sympatric oak species: *Quercus aliena*, *Q. dentata* and *Q. variabilis* at the northern edge of their distribution in China**

Jia Lyu^1^, Jia Song^1^, Yuan Liu^1^, Yuyao Wang^1^, Junqing Li^1^, Fang K. Du^1*^

*** Correspondence:** Fang K DU dufang325@bjfu.edu.cn

# Supplementary Figures and Tables

## Supplementary Figures


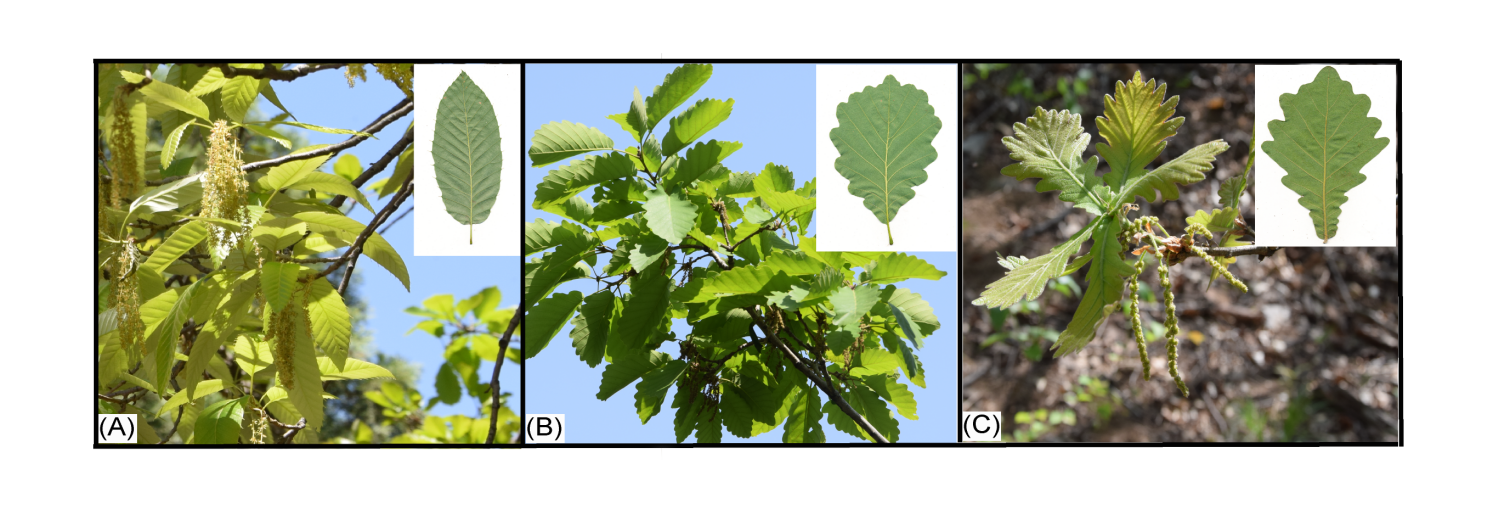


**Supplementary Figure 1.** Leaf photos of three oak species (A) *Q. variabilis,* (B) *Q. aliena* and (C) *Q. dentata*


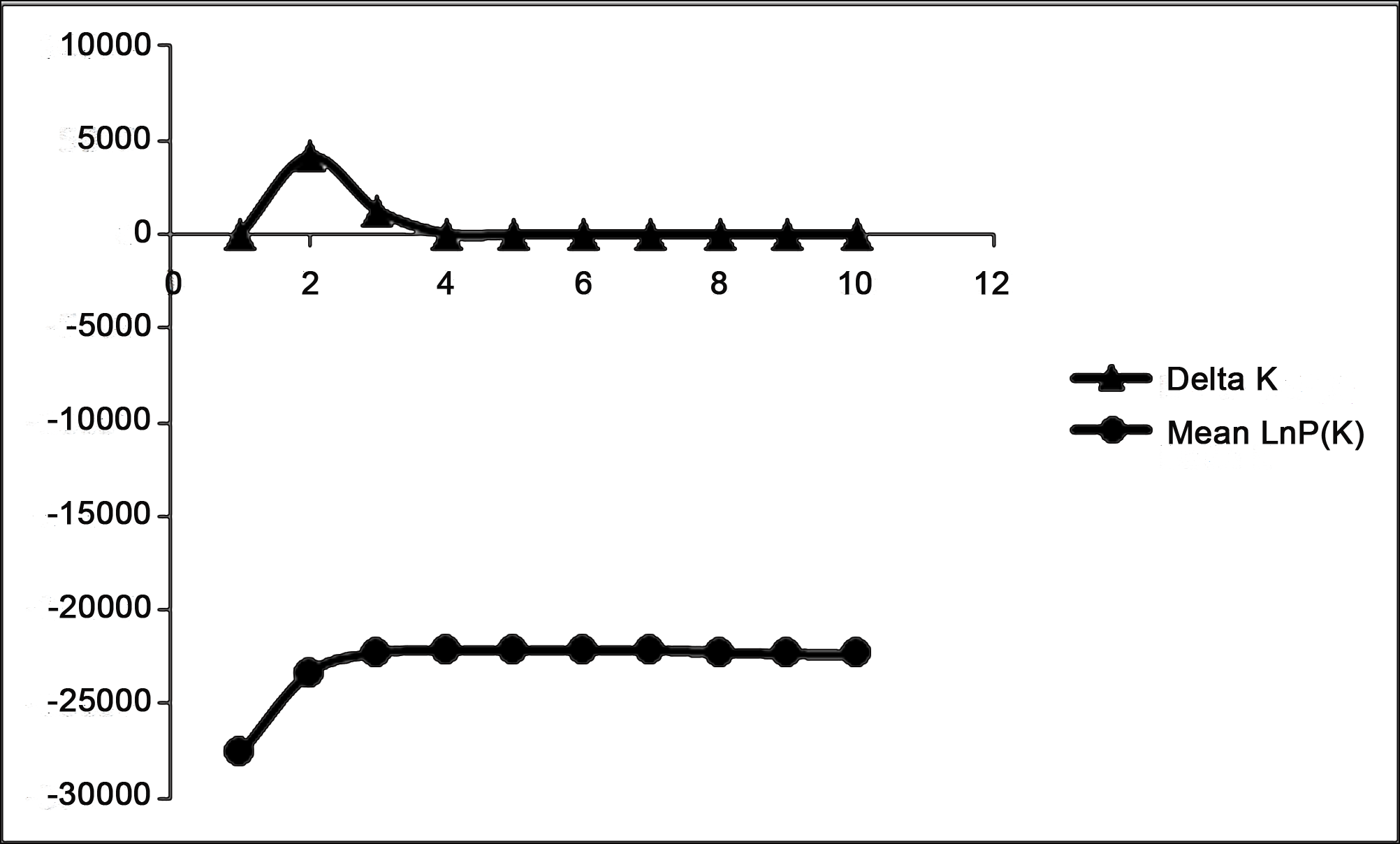


**Supplementary Figure 2.** Mean of LnP(K) of the data over 10 replicated runs (below) and Δ*K* (above).

# 1.2. Supplementary Tables

**Supplementary Table 1.** Summary information on sample sites, including site names, sample sizes for chloroplast DNA sequences and nuclear microsatellite (nSSRs) markers, altitude and geographical coordinates of each site

| Sites | cpDNA | nSSRs | latitude | longitude | altitude (m) |
| --- | --- | --- | --- | --- | --- |
| SHG | 30 | 33 | 39.6723 | 115.8073 | 283 |
| XSL | 116 | 125 | 39.6746 | 115.8233 | 621 |
| TK | 39 | 37 | 39.678 | 115.8303 | 509 |
| YSD | 49 | 58 | 39.6777 | 115.8169 | 496 |
| TL | 29 | 58 | 39.6795 | 115.8242 | 439 |
| ZL | 44 | 63 | 39.663 | 115.8304 | 562 |
| TZF | 36 | 40 | 39.6803 | 115.823 | 676 |
| Total | 343 | 414 | - | - | - |

**Supplementary Table 2.** Primer pairs used for amplification of the five intergenic spacer regions of chloroplast DNA.

| Region | Primer name and Sequence (5’ to 3’) | Reference | Sequence accession number |
| --- | --- | --- | --- |
| *psaI-accD* | **accD:** AATYGTACCACGTAATCYTTTAAA | Shaw et al., 2007 | KY751544 to |
|  | **psaI-75R:** AGAAGCCATTGCAATTGCCGGAAA |  | KY751546 |
| *trnH-psbA* | **trnH:** CGCGCATGGTGGATTCACAAT CC | Shaw et al., 2005 | KY751555 to |
|  | **psbA:** GTTATGCATGAACGTAATGCTC |  | KY751557 |
| *rpS12-rpL20* | **rpS12:** ATTAGAAANRCAAGACAGCCAAT | Shaw et al., 2005 | KY751547 to |
|  | **rpL20:** CGYYAYCGAGCTATATAT CC |  | KY751551 |
| *rpS16* | **rpS16F:** AAACGATGTGGTARAAAGCAAC | Shaw et al., 2005 | KY751552 to |
|  | **rpS16R:** AACATCWATTGCAASGATTCGATA |  | KY751554 |
| *trnQ-trnS* | **trnQ-F:** GCTCTGTATTTGTCTTAATTCTGC | Du et al., 2016 | KY751558 to |
|  | **trnS-R:** CCACATACCTTATTTTGACACCA |  | KY751561 |

**Reference**

Du, F.K., Hou, M., Wang, W.T., Mao, K.S., Arndt, H. (2016). Phylogeography of *Quercus aquifolioides* provides novel insights into the Neogene history of a major global hotspot of plant diversity in south-west China. J. Biogeogr. 44, 294–307. doi: 10.1111/jbi.12836

Shaw, J., Lickey, E.B., Beck, J.T., Farmer, S.B., Liu, W., Miller, J., et al. (2005). The tortoise and the hare II: relative utility of 21 noncoding chloroplast DNA sequences for phylogenetic analysis. Am. J. Bot. 92, 142–166. doi: 10.3732/ajb.92.1.142

Shaw, J., Lickey, E.B., Schilling, E.E., Small, R.L. (2007). Comparison of whole chloroplast genome sequences to choose noncoding regions for phylogenetic studies in angiosperms: the tortoise and the hare III. Am. J. Bot. 94, 275–288. doi: 10.3732/ajb.94.3.275

**Supplementary Table 3.** Details of the 27 pairs of microsatellite primers used for the initial amplification tests.

| locus | Primer Sequence (5’ to 3’) | Tm (°C) | Successfully amplification | motif | polymorphism | Ref | Selected in the paper |
| --- | --- | --- | --- | --- | --- | --- | --- |
| ssrQrZAG87 | TCCCACCACTTTGGTCTCTCA | 56 | No | — | — | Kampfer et al.1998 | No |
|  | GTTGTC AGCAGTGGGATGGGTA |  |  |  |  |  |  |
| MSQ13 | TGGCTGCACCTATGGCTCTTAG | 56 | Yes | (TC)nTTGCTGAA(TC)n | Yes | Dow et al.1995 | Yes |
|  | ACACTCAGACCCAQmCCCATTTTTCC |  |  |  |  |  |  |
| ssrQpZAG16 | CTTCACTGGCTTTTCCTCCT | 56 | No | — | — | Steinkellner et al.1997 | No |
|  | TGAAGCCCTTGTCAACATGC |  |  |  |  |  |  |
| PIE242 | TGGAGGGAAAAGAACAATGC | 56 | No | — | — | Durand et al.2010 | No |
|  | TTGCAATCCTCCAAATTTAATG |  |  |  |  |  |  |
| PIE227 | TACCATGATCTGGGAAGCAAC | 56 | Yes | TGG | Yes | Durand et al.2010 | Yes |
|  | AAGGGCTTGGTTGGGTTAGT |  |  |  |  |  |  |
| PIE271 | CACACTCACCAACCCTACCC | 56 | Yes | TC | Yes | Durand et al.2010 | Yes |
|  | GTGCGGTTGTAGACGGAGAT |  |  |  |  |  |  |
| PIE258 | TTCTCGATCTCAAAACAAAACCA | 56 | No | — | — | Durand et al.2010 | No |
|  | TTTGATTTGTTTAAGGAAAATTGGA |  |  |  |  |  |  |
| QmC00141 | ACACTACACTTAGCACCCCGCAA | 56 | No | — | — | Ueno et al. 2008 | No |
|  | GTTTCAATGGTAATCACCGCCTCCATA |  |  |  |  |  |  |
| QmC00196 | GTAACGGAATCGGAAAGGAGGTG | 56 | Yes | TAA | Yes | Ueno et al. 2008 | Yes |
|  | GTTTAGACTCAACCGTGCTGCTCTGAC |  |  |  |  |  |  |
| QmC00716 | AAGAGAACCCATTCCATCCCTGA | 56 | Yes | TC | Yes | Ueno et al. 2008 | Yes |
|  | GTTTCCCGAACAGTGGTTTCTTGA |  |  |  |  |  |  |
| QmC00822 | TTTGATCACAAAACAGAGGGACAGA | 56 | No | — | — | Ueno et al. 2008 | No |
|  | GTTTGCTCCTTGCTTAACAGGTGGAG |  |  |  |  |  |  |
| QmC00932 | AGGCTCAAAACAAAACCAAACCG | 56 | Yes | TC | Yes | Ueno et al. 2008 | Yes |
|  | GTTTCCCCTTTCCCATAATCAAACCCT |  |  |  |  |  |  |
| QmC00963 | TGAAGCCTGCGTTAACAACAACA | 56 | Yes | GAT | Yes | Ueno et al. 2008 | Yes |
|  | GTTTCTCCTCCTCCTCGCTACTCGT |  |  |  |  |  |  |
| QmC01758 | GGCACGAGGTTTCTTTGCTTAGAC | 56 | No | — | — | Ueno et al. 2008 | No |
|  | GTTTCCCACCAGTACTTTGGGCTTACC |  |  |  |  |  |  |
| QmC02052 | CACACCCAGATCCACAAAACTCC | 56 | Yes | AG | Yes | Ueno et al. 2008 | Yes |
|  | GTTTGCCTCTACGGTCTCCCTCTT |  |  |  |  |  |  |
| QmC02241 | TCAGTGACCACACGTCACCTCTC | 56 | No | — | — | Ueno et al. 2008 | No |
|  | GTTTCTTGGCCATGTTTTGATGG |  |  |  |  |  |  |
| DN950446 | TCTCTTTCTCCGTCCATTATCGC | 56 | Yes | AG | Yes | Ueno and Tsumura 2008 | Yes |
|  | GTTTCTCCACAGACCCCATTTCC |  |  |  |  |  |  |
| DN950726 | GCAAGAAGCATGCAGATGGAGAT | 56 | Yes | GAT | Yes | Ueno and Tsumura 2008 | Yes |
|  | GTTTGCATGGCCGTCATTAGCATTAAG |  |  |  |  |  |  |
| CcC00063 | CAATTGTACTCTGATGTCCCCGC | 56 | Yes | AGA | Yes | Ueno et al. 2009 | Yes |
|  | GTTTACCGGAGACTCAAAGAATCGACC |  |  |  |  |  |  |
| GOT011 | CCCCACCGTCTACTCTCAAA | 56 | Yes | TC | Yes | Durand et al. 2010 | Yes |
|  | GCGTTCACCACGTCCATAAT |  |  |  |  |  |  |
| GOT021 | AGAAAGTTCCAGGGAAAGCA | 56 | Yes | AT | Yes | Durand et al. 2010 | Yes |
|  | CTTCGTCCCCAGTTGAATGT |  |  |  |  |  |  |
| PIE163 | GAGAGGCATGTGGAACCAAG | 56 | Yes | AG | Yes | Durand et al. 2010 | Yes |
|  | CAAGCATAGGTGGTGGAACC |  |  |  |  |  |  |
| FIR026 | CTTCATGCACCAATTCCTCA | 56 | Yes | TC | Yes | Durand et al. 2010 | Yes |
|  | GGCCATGTATGTGTGCAAAA |  |  |  |  |  |  |
| WAG066 | AACCTGTTTGGCTTCGTGTG | 56 | Yes | AG | Yes | Durand et al. 2010 | Yes |
|  | AACAAAAGATTGGGAGGTGC |  |  |  |  |  |  |
| WAG068 | TCTGCAACAAAACCAAAACAC | 56 | Yes | AG | Yes | Durand et al. 2010 | Yes |
|  | CGGAGGAGAGAGTCAGCAAC |  |  |  |  |  |  |
| POR017 | CCCATATCCCTCTACGAAAGAA | 56 | Yes | CT | Yes | Durand et al. 2010 | Yes |
|  | CTGGAGATGACATAGTGTCTCAAA |  |  |  |  |  |  |
| FIR015 | ACCCTAAAACCCCAATCACC | 56 | Yes | AC | Yes | Durand et al. 2010 | Yes |
|  | CGGATCTTCGGCTATTCTTG |  |  |  |  |  |  |

**References**

Dow, B.D., Ashley, M.V., Howe, H.F. (1995). Characterization of highly variable (GA/CT)_n_ microsatellites in the bur oak, *Quercus macrocarpa*. Theor. Appl. Genet. 91, 137-141. doi: 10.1007/BF00220870

Durand, J., Bodénès, C., Chancerel, E., Frigerio, J.M., Vendramin, G., Sebastiani, F., et al. (2010). A fast and cost-effective approach to develop and map EST-SSR markers: oak as a case study. BMC Genomics 11, 570. doi: 10.1186/1471-2164-11-570

# Ueno, S., [Tsumura](https://www.researchgate.net/profile/Yoshihiko_Tsumura2), Y. (2008). Development of ten microsatellite markers for *Quercus mongolica* var. *crispula* by database mining. [Conserv. Genet](https://www.researchgate.net/journal/1566-0621_Conservation_Genetics). 9, 1083-1085. doi: 10.1007/s10592-007-9462-4

Ueno, S., Taguchi, Y., Tsumura, Y. (2008). Microsatellite markers derived from *Quercus mongolica* var. *crispula* (Fagaceae) inner bark expressed sequence tags. Genes Genet. Syst. 83, 179-87. doi: 10.1266/ggs.83.179

Ueno, S., Aoki, K., Tsumura, Y. (2009). [Generation of Expressed Sequence Tags and development of microsatellite markers for](https://www.researchgate.net/publication/43178544_Generation_of_Expressed_Sequence_Tags_and_development_of_microsatellite_markers_for_Castanopsis_sieboldii_var_sieboldii_Fagaceae) *[Castanopsis sieboldii](https://www.researchgate.net/publication/43178544_Generation_of_Expressed_Sequence_Tags_and_development_of_microsatellite_markers_for_Castanopsis_sieboldii_var_sieboldii_Fagaceae)* [var.](https://www.researchgate.net/publication/43178544_Generation_of_Expressed_Sequence_Tags_and_development_of_microsatellite_markers_for_Castanopsis_sieboldii_var_sieboldii_Fagaceae) *[sieboldii](https://www.researchgate.net/publication/43178544_Generation_of_Expressed_Sequence_Tags_and_development_of_microsatellite_markers_for_Castanopsis_sieboldii_var_sieboldii_Fagaceae)* [(Fagaceae)](https://www.researchgate.net/publication/43178544_Generation_of_Expressed_Sequence_Tags_and_development_of_microsatellite_markers_for_Castanopsis_sieboldii_var_sieboldii_Fagaceae). [Ann. For. Sci](https://www.researchgate.net/journal/1286-4560_Annals_of_Forest_Science). 66, 509. doi: 10.1051/forest/2009037

**Supplementary Table 4.** Ten different migration models used in Migrate-n.

| Number | Model | Species and model parameters | | |
| --- | --- | --- | --- | --- |
|  |  | *Q. variabilis* | *Q. aliena* | *Q. dentata* |
| 1 | Full | *** | *** | *** |
| 2 | nearly no gene flow between *Q. variabilis* and either *Q. aliena* or *Q. dentata* | *ccª | c** | c** |
| 3 | no gene flow from *Q. variabilis* to either *Q. aliena* or *Q. dentata* | *** | 0** | 0** |
| 4 | no gene flow from either *Q. aliena* or *Q. dentata* to *Q. variabilis* | *00 | *** | *** |
| 5 | no gene flow from *Q. aliena* to *Q. variabilis* | *0* | *** | *** |
| 6 | no gene flow from *Q. dentata* to *Q. variablis* | **0 | *** | *** |
| 7 | no bidirectional gene flow between *Q. aliena* and *Q. variabilis* | *0* | 0** | *** |
| 8 | no bidirectional gene flow between *Q. dentata* and *Q. variabilis* | **0 | *** | 0** |
| 9 | no gene flow from *Q. variabilis* to *Q. aliena* | *** | 0** | *** |
| 10 | no gene flow from *Q. variabilis* to *Q. dentata* | *** | *** | 0** |
| a: migration between *Q. variabilis* and either *Q. aliena* or *Q. dentata* was set to c (0.01) instead of 0 to meet the needs with regard to coalescence trees of Migrate-n. | | | | |

**Supplementary Table 5.** Chloroplast DNA sequence variations.

| Haplotype | Nucleotide position | | | | | | | | | | | | | | | | | | | | | | | | | | | | | |
| --- | --- | --- | --- | --- | --- | --- | --- | --- | --- | --- | --- | --- | --- | --- | --- | --- | --- | --- | --- | --- | --- | --- | --- | --- | --- | --- | --- | --- | --- | --- |
|  | *psaI-accD* | | | | | | |  | *trnH*-*psbA* | | | | | |  | *rpS12-rpL20* | | | | | |  | *rpS16* | | | | | |  | *trnQ-trnS* |
|  | 2 | 3 | 3 | 5 | 5 | 5 | 6 |  |  |  | 2 | 3 | 3 | 4 |  | 1 | 1 | 4 | 6 | 7 | 7 |  |  | 2 | 3 | 5 | 6 | 7 |  | 2 |
|  | 1 | 2 | 5 | 0 | 8 | 8 | 7 |  | 4 | 8 | 4 | 6 | 9 | 9 |  | 5 | 7 | 5 | 1 | 0 | 9 |  | 1 | 7 | 5 | 1 | 7 | 6 |  | 4 |
|  | 3 | 4 | 9 | 6 | 5 | 6 | 8 |  | 6 | 5 | 4 | 9 | 4 | 2 |  | 5 | 8 | 2 | 9 | 4 | 8 |  | 3 | 2 | 6 | 6 | 2 | 0 |  | 4 |
| H1 | C | T | G | G | C | ﹢ | ﹣ |  | T | T | C | ﹣ | ﹣ | T |  | A | T | A | ﹣ | T | A |  | G | Δ | A | A | A | G |  | C |
| H2 | A | C | A | T | A | ﹣ | * |  | C | C | T | Φ | ω | G |  | A | A | G | λ | G | T |  | T | ﹣ | G | C | C | A |  | G |
| H3 | A | C | A | T | A | ﹣ | * |  | C | C | T | Φ | ω | G |  | T | A | G | λ | G | T |  | T | ﹣ | G | C | C | A |  | G |
| H4 | C | T | G | G | C | ﹢ | ﹣ |  | T | T | C | ﹣ | ﹣ | T |  | T | T | A | ﹣ | T | A |  | G | Δ | A | A | A | G |  | C |
| H5 | C | T | G | G | C | ﹢ | ﹣ |  | T | T | C | ﹣ | ﹣ | T |  | A | A | G | λ | G | T |  | G | Δ | A | A | A | G |  | C |
| H6 | A | C | A | T | A | ﹣ | * |  | C | C | T | Φ | ω | G |  | A | A | G | λ | G | T |  | T | ﹣ | G | C | C | A |  | C |
| H7 | A | C | A | T | A | ﹣ | * |  | C | C | T | Φ | ω | G |  | A | A | G | ﹣ | T | T |  | T | ﹣ | G | C | C | A |  | G |

+: TTATTATATCACATATACTCACTTC

*: ATAACAATATAA

Φ: ATTTAATA

ω: TTAATAAA

λ: TTTA

Δ: AATTTA
